# Printed Strain Gauge on 3D and Low-Melting Point Plastic Surface by Aerosol Jet Printing and Photonic Curing

**DOI:** 10.3390/s19194220

**Published:** 2019-09-28

**Authors:** Michela Borghetti, Mauro Serpelloni, Emilio Sardini

**Affiliations:** Department of Information Engineering, University of Brescia, 25123 Brescia, Italy

**Keywords:** printed electronics, aerosol jet printed, photonic sintering, strain gauges, non-planar surfaces, plastics, temperature sensitive, silver ink

## Abstract

Printing sensors and electronics directly on the objects is very attractive for producing smart devices, but it is still a challenge. Indeed, in some applications, the substrate that supports the printed electronics could be non-planar or the thermal curing of the functional inks could damage temperature-sensitive substrates such as plastics, fabric or paper. In this paper, we propose a new method for manufacturing silver-based strain sensors with arbitrary and custom geometries directly on plastic objects with curvilinear surfaces: (1) the silver lines are deposited by aerosol jet printing, which can print on non-planar or 3D surfaces; (2) photonic sintering quickly cures the deposited layer, avoiding the overheating of the substrate. To validate the manufacturing process, we printed strain gauges with conventional geometry on polyvinyl chloride (PVC) conduits. The entire manufacturing process, included sensor wiring and optional encapsulation, is performed at room temperature, compatible with the plastic surface. At the end of the process, the measured thickness of the printed sensor was 8.72 μm on average, the volume resistivity was evaluated 40 μΩ∙cm, and the thermal coefficient resistance was measured 0.150 %/°C. The average resistance was (71 ± 7) Ω and the gauge factor was found to be 2.42 on average.

## 1. Introduction

Printed electronics (PE) is one of the most fasten growing sectors for developing sensors and electronics over the last few decades [1]. It generally consists of the deposition of functional inks or pastes over various substrates by means of a set of techniques (screen printing, flexography, gravure printing, inkjet printing, aerosol printing, etc.) [2]. The functional materials can be organic or inorganic [3] and they can have conductive, semiconductive, or dielectric properties such as metal nanoparticles (gold, silver, copper, carbon, etc.) [4], polymer semiconductors (regioregular poly(3-hexylthiophene) (P3HT), poly (triarylamine), etc.) [5] or insulting polymers (polydimethylsiloxane, polyimide, etc.) [6], as well as being biocompatible, biodegradable, or recyclable [7]. The typical substrates used for PE are plastics [8], fabric [9], glass [10], with different mechanical properties such as flexibility, bendability and stretchability [11]. Since a large variety of materials can be adopted, PE can overcome some limitations of the silicon-based electronics, especially in flexible electronic applications such as flexible electrodes for healthcare applications [9], wearable devices [12], or bioelectronic applications by using organic semiconductors [13] which better match mechanical and conduction properties of biotic tissue. Furthermore, since it offers low-cost processes for large areas and flexible applications [14] with minimum material wastage and reduced time consumption [15], PE attracts a growing interest in producing light devices such as flexible displays, electrodes, sensors, antennas, radio-frequency identification tags, and solar cells [16]. In the field of the Internet of Things (IoT), where everyday objects are equipped with sensing, computing, and storage capabilities that communicate information [17], PE promotes the development of customized IoT devices equipped with fully or hybrid advanced printed electronics, of arbitrarily complex pattern, and tailor-made for a specific application [12]. For example, Falco et al. proposed a printed smart RFID tag designed as a wireless sensor network able to provide both light intensity and temperature information and oriented for IoT applications [18]. In the field of Industry 4.0, additive manufacturing (AM) is crucial to create and customized prototypes or products of arbitrary shape for reducing lead time, costs, and material wastage [19]. The combination of AM and PE for fabricating smart objects has attracted more attention in recent years [20,21] because printing electronics directly on an object’s surface could take advantage of the PE (low-cost, fabrication flexibility, etc.) and could avoid any installation/assembly and the associated problems [19,21]. Indeed, the installation of electronics and sensors often includes the surface preparation (cleaning, scratching, etc.) and the deposition of an adhesive layer with proper and specific characteristics. Furthermore, when wrapping flexible sheets, wrinkles and folds can formed [20]. In the case of sensors, the adhesive layer interferes with the proper functioning of the sensor [22,23] and, if the sensor is not properly attached to the surface, the risk of detachment increases, or the sensor does not work properly. Few printed electronic methods are suitable for printing over non-planar or 3D surfaces. Wang et al. have recently proposed a cut-and-paste method to produce electronic tattoos [24] for noninvasive wearable devices mounted on skin. Water Transfer Printing (WTP) technology is a new promise method to transfer planar electronics to 3D surfaces [20,25], but more investigation is required to control and improve the positioning and the size of the printed tracks, as well as to establish the resolution and the accuracy of WTP. Among the methods based on direct printing of electronics on 3D surfaces, laser direct structuring (LDS) is a valuable solution to deposit conductive tracks (made of gold, copper, or nickel) selectively on a large variety of plastics, but the system requires specific safety measures and only limited materials can be deposited [26]. A new printed method that can be used for fabricating PE directly over a non-planar or 3D surface is the Aerosol Jet Printing (AJP) technique introduced by Optomec Inc. [27]. APJ is a non-contact printing method; therefore, expensive masks are not required. It consists of the deposition of functional inks atomized in an aerosol gas which passes through a nozzle. With respect to other printing methods, the viscosity of inks is in the large range from 0.5 to 2000 cP. The high resolution (lower than 10 μm) and the printing quality make AJP a valuable method to produce devices with increased geometric complexity [28] and for a wide variety of applications. Furthermore, the distance between the nozzle and the surface of about 5 mm allows the deposit of the ink on 2D and 3D surfaces easily [29]. In the literature, some examples are reported [27,28,29,30,31,32,33]. Examples of metallization of photovoltaics are described in [27,30]. In [31], AJP was investigated to increase the aspect ratio of printed lines for the fabrication of current-carrying microcircuits with a high density of elements. In [32], a humidity sensor was realized over the surface of a packaged integrated circuit for demonstrating the good integration with printed and silicon-based electronics. The interdigitated nano-particle silver electrodes were deposited with APJ while the sensitive layer of Nafion® was drop cast over the electrodes. In [33], a six-electrochemical-sensors platform with microfluidics was realized entirely with APJ to quantify glucose at different concentrations using a standard enzyme-mediated procedure. 

As shown above, the printing process is fundamental, but it is not enough to guarantee an optimal PE manufacturing. A proper curing process of the printed layer is required to activate the functional properties of the ink. During the process, the printed device is heated in order to drive evaporation of the carrier solvent and of the other additives and to form a solid mass of material. In the case of conductive layer, the sintering process is crucial for achieving the proper conductivity and guaranteeing the proper functionality of the device. However, a prolonged and high-temperature process required for sintering metallic nanoparticles is not compatible with some substrates, such as some polymers. For example, while the cure temperature of the silver nanoparticles is higher than 150 °C and the cure time is higher than 30 min, the melting point of the polyvinyl chloride (PVC) is about 100 °C. For example, in [34], AJP was used to fabricate metal strain gauges depositing directly on planar surfaces, but the curing process was performed in a furnace at 350°. Photonic sintering [35] is a valid method to sinter conductive inks, because it heats the outer layers (few micrometers) with high-density energy in a short time and prevents the overheating of the substrate. A flash lamp dries and sinters the printed layer for a short exposure time (few millisecond) and illuminates a large area (up to 150 × 4800 mm) at the same time, reducing the curing time. In [36], photonic sintering was used for curing thermoelectric films deposited on paper, polyimide and glass. In [37], copper electrodes were fabricated by inkjet printing method and photonic sintering on a benzocyclobutene polymer layer; these electrodes were used as source and drain electrodes in an organic thin-film transistor.

In the literature, since they are relatively new, AJP or photonic sintering are still little used and usually used separately. A preliminary analysis was evaluated in [38]. In this paper, we propose to combine AJP and photonic sintering as a promising method for the manufacturing strain sensors at environmental temperature on non-conventional surfaces (such as flexible, non-planar, or 3D, with a low melting point, etc.). The entire process, including wiring and encapsulation, is performed at room temperature. For the validation of the proposed manufacturing method, we designed, manufactured, and tested strain gauges with conventional geometry on PVC conduit with the proposed method. Strain gauges are simple and well-known sensors, commonly used for measuring strains triggered by forces and torques on the object. Printing strain gauges with conventional geometry on cylindrical surface simplifies the comparison with commercial strain sensors, and could demonstrate the feasibility of the proposed method for manufacturing printed sensors and electronics when conventional technologies are not suitable to embed custom electronics (with unique or complex pattern) in 3D objects. For example, printed strain gauges can be useful for structural health monitoring [39,40], and predictive maintenance. These two aspects are very attractive in smart factories since they help to prevent failure or defects on the production system, monitoring the current state of system health and determining when maintenance should be performed. 

## 2. Materials and Methods

### 2.1. Materials and Strain Sensor Geometry

In this work, a commercial silver nanoparticle ink (Metalon® HPS-108AE1), commercialized by Novacentrix (Austin, TX, US) and suitable for AJP, was used as received. This ink consists on silver nanoflakes (of 0.4–0.7 μm diameter) dispersed in a water-based liquid and it includes a urethane additive for promoting the adhesion of the ink to plastic surfaces. The declared viscosity of ink is 130–180 cP, compatible with the specifications of the APJ system.

We decided to deposit the ink on plastic and non-planar surfaces since the plastic is largely used to produce any kind of object, whose surface is usually non-planar. Furthermore, some plastics are sensitive-temperature materials incompatible with traditional curing methods that expose the object to temperatures (>150 °C) higher than their melting point (*T_m_*) and their glass transition temperature (*T_g_*) for a long time. In this work, we tested the manufacturing process by depositing the silver ink on substrates made of plastic materials commonly used to produce commercial or industrial objects: Polyethylene terephthalate (PET, *T_g_* = 70 °C, *T_m_* = 250 °C), polyethylene (*T_g_* = −78 °C, *T_m_* = 120 °C), and PVC (*T_g_* = 70 °C, *T_m_* = 100 °C); in particular, PVC is a very appealing plastics for its low-cost and its excellent properties [41]. We also selected the thermoplastic polymer polylactic acid (PLA, *T_g_* = 70 °C, *T_m_* = 150 °C), since it is largely used in addictive manufacturing for its excellent biocompatibility and sustainability [42]. We also selected polyimide (PI, *T_g_* = 360 °C), because it is a valuable material used to produce flexible electronics. After verifying the great adhesion of the silver to all selected plastic materials, we selected a PVC conduit (*T_g_* = 70 °C, *T_m_* = 100 °C) with Young modulus of 3.7 GPa and yield strain of 0.6%, in order to extensively evaluate the electrical properties of the final printed device and to demonstrate the feasibility of the manufacturing process for producing and integrating electronics and sensors on plastic objects, with curvilinear surfaces. The outer diameter of the conduit is 20 mm and the inner diameter is 17 mm. Even if it has a simple geometry, the non-planar and plastic surface of the conduit represents a challenge for the most of PE methods and it could represent a good starting point for producing electronics and sensors on more challenging surfaces. 

**Figure 1:**
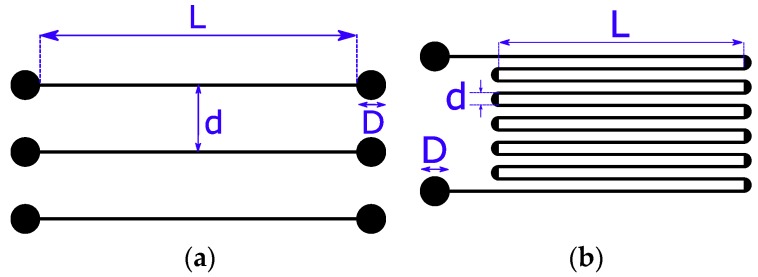
Geometric pattern of the printed devices adopted to test the proposed method: (**a**) Three lines 3.5 mm apart (d), 13 mm long (L) with 1.2 mm diameter pads (D); (**b**) serpentine pattern with lines 10 mm long (L) and 1.2 mm diameter pads (D).

The geometric patterns of the printed devices are shown in Figure 1. The three stripes (pattern shown in Figure 1a) were used to analyze and tune AJP and photonic sintering parameters. The serpentine pattern of Figure 1b was used to fabricate strain gauges deposited directly on the conduits. The pattern is a grid of straight segments and circular arcs; the width of each segment corresponds to the width of one single printed line, which depends on the used materials and on the control parameters of the printing process (printing speed, nozzle size, number of deposited layers, etc.). The serpentine pattern has a simple geometry typically used in commercial foil strain sensors, but it represents a preliminary study of a new manufacturing process for producing more complex and customized sensors and electronics directly on the object. The experimental results on the characterization of the produced sensor simplify the comparison with the commercial strain sensor.

CircuitWorks Conductive Epoxy CW2400 was used to make the electrical contact between the printed pads and wires. It is a two-parts silver epoxy with volume resistivity lower than 1 mΩ/cm and it cures in contact with air at room temperature. 

Furthermore, we tested a protective coating (SG250) produced by HBM (Darmstadt, Germany) to encapsulate and protect the printed layer. This silicone rubber is one-component covering material typically used for strain gauge installation. It is a transparent silicone rubber, solvent-free and it cures in contact with air at room temperature. This protective layer should not stiffen the covered area and should not damage the surface with solvents or heat. 

### 2.2. Manufacturing Process

**Figure 2:**
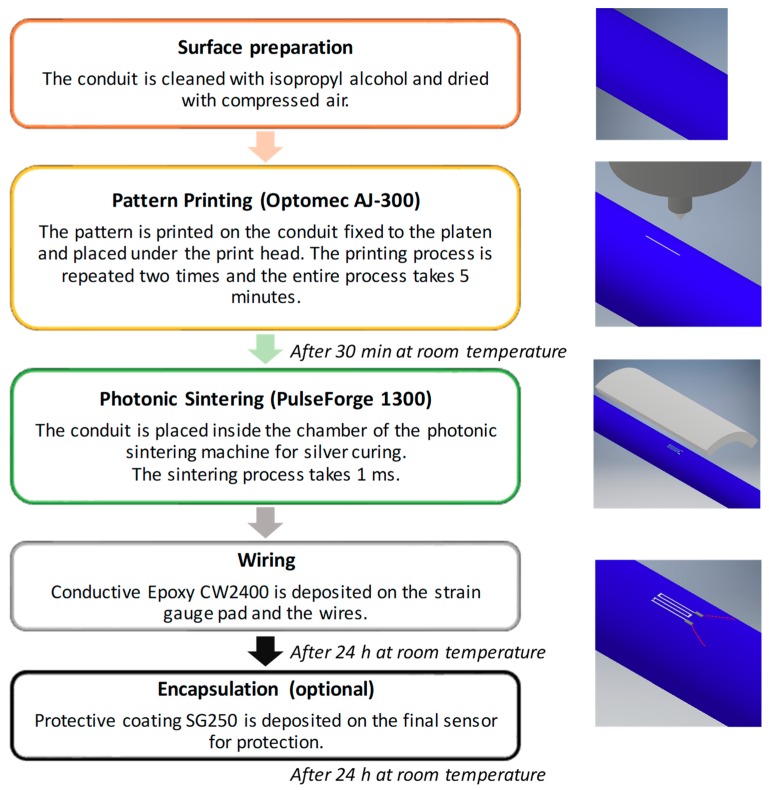
Manufacturing process for a strain gauge printed directly on a conduit. All the phases are performed at room temperature.

In Figure 2, the manufacturing process for the production of a strain gauge embedded in a PVC conduit is shown. All the phases are performed at room temperature. Before printing the sensor or the electronic circuit, it is recommended to clean the surface with the proper solvent. In the case of the PVC conduit, the surface was cleaned using isopropyl alcohol and dried using compressed air. Then, the conduit is placed under the print head in order to print the silver-based sensor. After that, the conduit is left to cure at room temperature for 30 min to permit the evaporation of the solvents. Then, the silver layer is cured with photonic sintering. Finally, a conductive epoxy is deposited for sensor wiring and it is left to cure at room temperature for 24 h. 

#### 2.2.1. Aerosol jet Printing

In order to print the silver ink directly on the PVC conduit, the Optomec AJ-300 was used. A 3D CAD geometry was prepared to program the 3D movement of print head and platen. According to ink viscosity, we adopted a pneumatic collision atomizer to transform the ink into an aerosol mist. The ink stirred inside a jar is atomized by the flow of nitrogen gas (carrier gas) and pushed towards the print head. Since the mist is quite dilute, the removal of the superfluous carrier gas occurs inside the virtual impactor, which also adjusts the droplet size of the aerosol by regulating the exhaust gas. The diameter of the droplets outside coming out the virtual impactor is between 1 to 5 microns. The resulting mist reaches the print head, where the aerosol is focused by a nitrogen sheath gas. The sheath gas collimates the beam in order to prevent the nozzle clogging and to reduce the radius of aerosol up to a tenth of the nozzle diameter; indeed, the sheath gas surrounds the beam insulating the inner walls from the ink droplet. Finally, the aerosol beam reaches the object/substrate passing through the nozzle. The substrate is fixed to a movable and vacuum platen, controlled according to the geometry to be printed. The nitrogen gas was used because it is an inexpensive and inert gas. The flow rate of the carrier gas, of the exhaust gas and of the sheath gas contributes to regulate the quantity of the deposited material and thus the thickness and the width of the deposited lines. The system is able to deposit inks with viscosity between 1 to 1000 cP. According to the ink rheology adopted in this work, we set the following flow rate values: 1100 sccm for the carrier gas, 1020 sccm for the exhaust gas, and 150 sccm for the sheath gas. We selected a nozzle of 300 μm diameter. The printing speed of the platen was 1 mm/s and the distance between the nozzle and the substrate was 4 mm. According to desired electrical characteristics, the process can be repeated several times, depositing several ink layers before curing without misalignments. The declared position accuracy and the position repeatability provided by the manufacturer are lower than ±5 μm, which allows to reach a line width of 10 μm. The system is able to print on surfaces inclined of up to 70 degrees without tilting the print head, as declared by the manufacturer. After printing, the object was placed at environmental temperature for 30 min for the drying of the solvent and of the additives.

#### 2.2.2. Photonic Sintering

**Figure 3:**
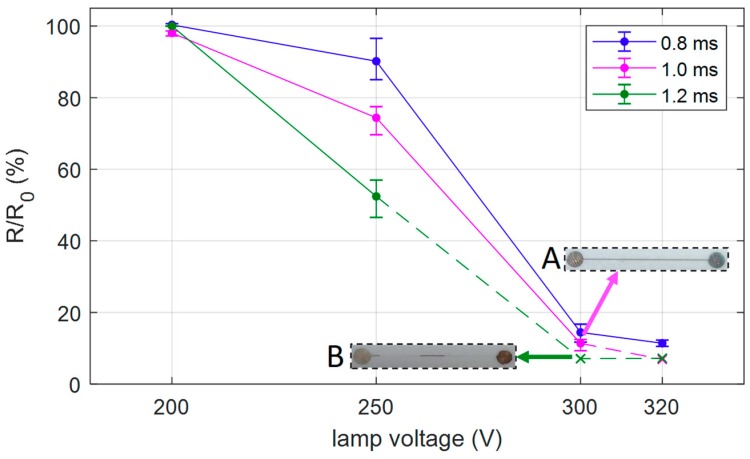
Ratio between the resistance measured after (*R*) and before (*R*_0_) the sintering process. Each point is the average ratio of the stripes sintered with the same process and the error bar is their span. The inset A shows the aspect of the stripe after the sintering process with the best setting (lamp voltage = 300 V, pulse width = 1.0 ms). The cross symbol indicates that some lines were damaged during the process. If the line is damaged (inset B), the resistance is considered infinite and it is not included in the average calculation.

The curing of the deposited material was performed with PulseForge 1300, a photonic sintering machine commercialized by Novacentrix. This tool enables the rapid sintering of the metallic ink preserving the plastic substrate. The system uses a broad-spectrum pulse of light emitted by a xenon flash lamp to heat the object placed on the plate inside the chamber. Pulse width and voltage of the flash lamp are two control parameters of the process that regulate the energy density irradiating the ink and their values were chosen accordingly to selected ink and substrate. An experimental investigation of the influence of the voltage profile of the lamp on the final printed device is required to find the best setting that guarantee good electrical (conductance) and mechanical (adhesion) properties of the printed device. We produced 12 samples on a PVC substrate with the proposed APJ method and each sample was exposed to one light pulse with a defined profile. Each sample consists of three silver lines according to the pattern shown in Figure 1a. The impulse width and the lamp voltage were varied in the range 0.8–1.2 ms and 200–320 V, respectively, for a total of 12 profiles. We measured the resistance of the stripes before (*R*_0_) and after (*R*) the curing process. The results are shown in Figure 3, the points represent the average value *R*/*R*_0_ of three stripes of one sample and the error bar includes all the three ratios. The damaged lines after the process were not considered for the calculation of average and span. 

Increasing voltage and impulse width, the resistance decreases, as expected, until the detachment of the stripe from the substrate (inset B). We selected a lamp voltage of 300 V and a pulse width of 1.0 ms as the best setting of the sintering process because it guarantees the lowest electrical resistance using less energy and without damaging the printed layer. In this case the resistance reaches the 11% of the starting value on average.

Adhesion of printed layers is crucial to ensure reliable mechanical and electrical performance. We tested the adhesion of the printed silver layers to different plastic surfaces: PVC, PET, PI, polyethylene, and PLA; we adopted the cross-cut tape test described in the standard ISO 2409 [43] for the evaluation of the adhesion. Cuts were made in vertical and horizontal directions of the pattern with a single-blade cutting tool, a 3M tape is applied onto the grid and then removed manually. In all the cases, we observed completely smooth edges of the cuts and none of the squares of the lattice was detached. On the contrary, without sintering process, the ink adhesion was very poor.

#### 2.2.3. Electrical Contacts and Encapsulation

In order to create the electrical contact between the printed device and the wires, we deposited manually a layer of the CW2400, which was cured at room temperature and humidity for 24 h; in this way, the wiring of the circuit is performed at room temperature and the entire fabrication process is suitable even for temperature-sensitive materials like plastics.

**Figure 4:**
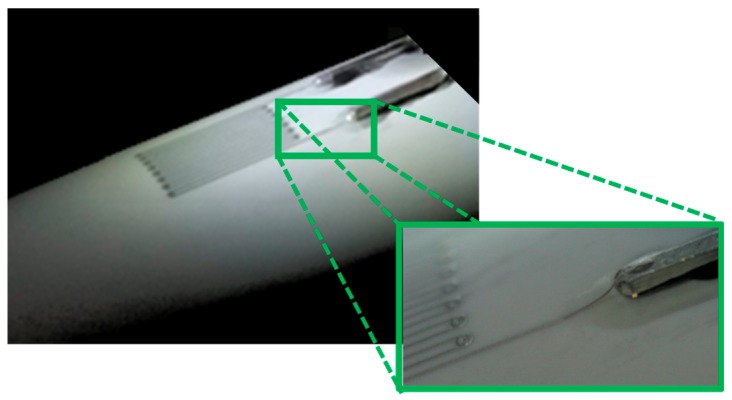
Strain gauge printed on a PVC conduit of 20 mm external diameter. The two ends of the printed serpentine are deposited on the right angle pin header connector.

Another way to connect the sensor and an external device with the proposed method is shown in Figure 4. In this case, the pads were not printed, and the ends of the printed sensor were deposited over the metallic pins of the connector fixed to the conduit, without deteriorating the electrical contact.

In some cases, we deposited the SG250 manually to encapsulate the printed device and we dried it at room temperature for one day.

## 3. Results and Discussion

**Figure 5:**
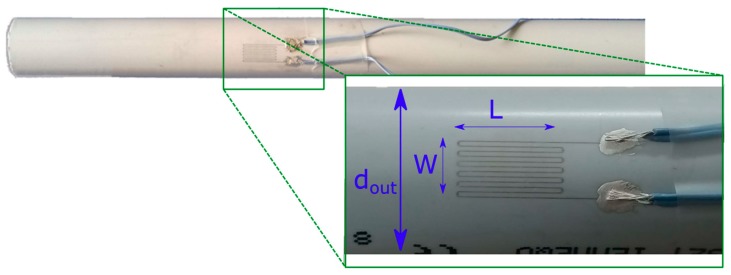
Strain gauge printed on a PVC conduit of 20 mm external diameter (d_out_). The active gauge length (L) is 10 mm and the gauge width (W) is 5.5 mm. Two wires connect each pad to the measurement system.

In order to analyze the behavior of the strain gauges realized with the proposed method, strain gauges (Figure 5), whose the pattern is shown Figure 1b, was printed on PVC conduits according to the process proposed in Section 2. To reach the proper electrical resistance, we repeated the printing process two times. During all electrical tests, the strain sensor resistance was measured by a HP34401 multimeter (Agilent, Santa Clara, CA, US) configured for 4-wire ohms measurements. In this configuration, the lead resistance introduced by the sensor and by the multimeter could be considered negligible. The multimeter was controlled by custom LabVIEW Virtual Instruments (VIs) for setting the multimeter and collecting and saving the raw measurements.

### 3.1. Thickness and Resistivity Measurements

An estimation of the resistivity of silver lines after the manufacturing process was performed to evaluate strain gauge properties. According to the second Ohm’s law, resistivity ρ can be expressed as in Equation (1):

(1) $$\rho =\frac{R\cdot l}{S},$$

where *R* is the resistance of the device, *l* is the overall length, and *S* is the cross section. 

**Figure 6:**
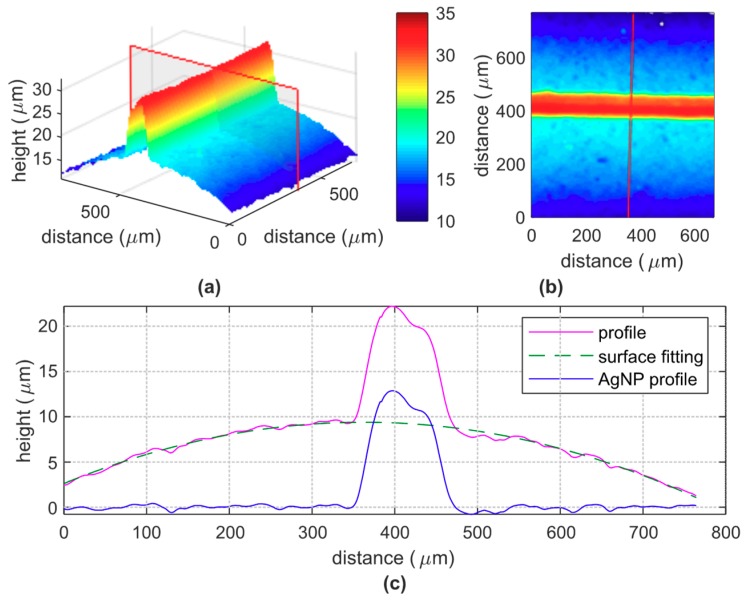
Profile of a portion (0.600 × 700 mm) of one segment of a serpentine device. (**a**) 3D view and plane to determine the cross-section size; (**b**) 2D view and line to determine the cross-section size; (**c**) cross section (magenta line) of the surface (conduit and sensor), with the fitted surface (attributable to the conduit surface) approximated to an arc of circle (green line) and the resulted profile of the only printed segment (blue line).

The thickness of the deposited ink and the width of the segments were measured by using an optical profilometer Profilm3D, commercialized by Filmetrics. The printed serpentines have a total length of 179 mm. The elaborated profile of a portion (0.600 × 700 mm) of one segment is shown in Figure 6a,b. The profile shown in Figure 6c is a cross section of the acquired surface with the plane confined by the red line (Figure 6a). Since the serpentine is printed on a cylindric surface, we fitted the points attributable to the conduit surface; the radius of the arc of circle (green line) is 9.8 mm in accordance with the measured radius of the conduit (10 mm). The profile of the printed line can be obtained by subtracting the measured profile with the fitted conduit surface (blue line). The maximum thickness is 12.87 μm and the width is 118 μm. The analysis was repeated 15 times on each sample, and we estimated a maximum thickness of 12.92 ± 0.26 μm and a width of 120 ± 6 μm. The average thickness is 8.72 μm. The resistivity calculated from Equation (1) is 40.0 ± 1.5 μΩ∙cm, considering an average cross section of the printed serpentine 1.05 nm^2^ and a resistance of 68 Ω; this result is in accordance with the datasheet of the silver ink. In accordance to the profile measurement on the printed stripe, the resolution of the manufacturing process is estimated around ±6 μm.

### 3.2. Sensor Response over Temperature

The PVC conduit equipped with the printed strain gauge was placed inside a climatic chamber Perani UC 150/70 to estimate the thermal coefficient resistance (TCR) of the deposited ink. A Pt100 was fixed on the PVC conduit closed to the strain sensor to measure the environment temperature around the sensor. The Pt100 and the strain sensor were connected each to a HP34401 multimeter configured for 4-wire ohms measurements. Multimeters and climatic chamber were controlled by a custom LabVIEW software permitting to automate and synchronize the acquisition process. The climatic chamber was pre-cooled at 10 °C before the characterization test. The temperature was increased from 10 to 45 °C (heating phase), with step of 5 °C, every 40 min. The temperature was decreased in the same way (cooling phase) and the cyclic test was repeated five times. The measured humidity inside the chamber was 15% ± 2% for all the test.

**Figure 7:**
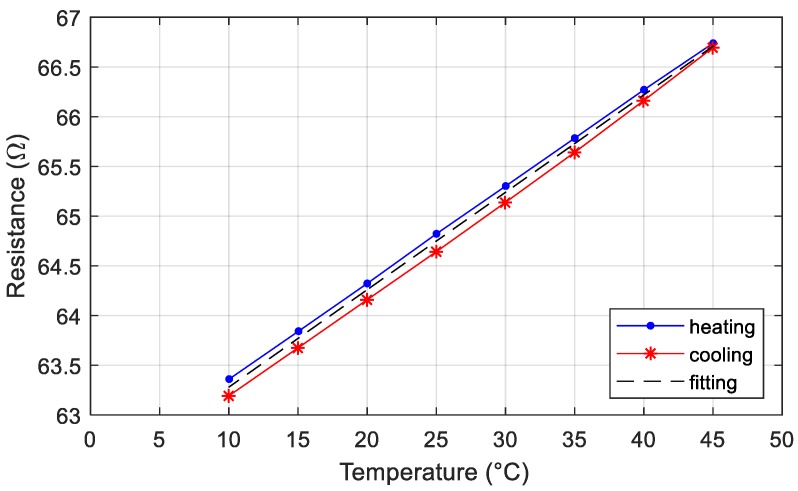
Resistance of the strain sensor printed on a PVC conduit over temperature. The blue line is obtained by increasing the temperature, whereas the red one is obtained by decreasing the temperature. The black dashed line best fits five repeat experiments and it is calculated by using the linear least squares (LLS) method.

The experimental results are summarized in Figure 7. Each data point is the average value calculated on the five cycles and the maximum absolute deviation is 0.1 Ω in the worst case. The thermal coefficient of resistance (TCR) is 0.150 %/°C and it is half of the bulk silver. We considered the TCR as the slope of the line that best fits all the data collected in the five repeated tests and it is normalized on the minimum measured resistance. This fitting line is obtained by using the linear least squares (LLS) method. The resistance hysteresis is 4.9% of the full-scale range. 

### 3.3. Sensor Response under Mechanical Uniaxial Stress

**Figure 8:**
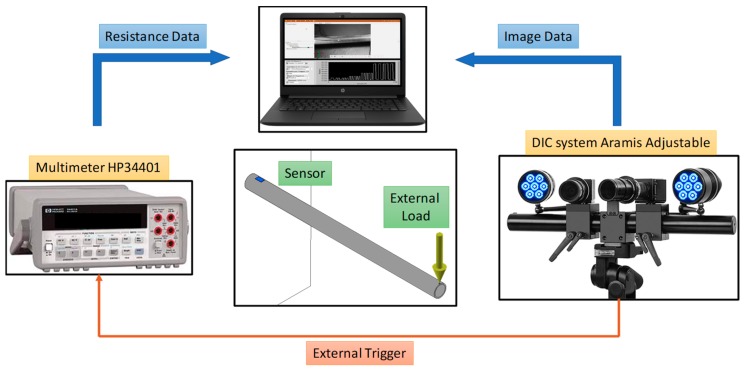
Experimental setup for the characterization of a strain gauge printed on a conduit. The DIC system records the deformation in the conduit area including the sensor, while the multimeter measures the electrical resistance of the sensor. A notebook collects and controls the two instruments. The acquisition of the resistance is triggered on the ACQUISITION of the images taken by DIC system. The conduit is anchor at the end where the sensor is printed, while external loads are applied on the free end to induce a strain.

The printed strain gauges were tested by using the experimental setup shown in Figure 8. 

The conduit was anchored at one end and several weights were applied on the free end. In this way the conduit could be considered as a cantilever beam under uniaxial stress. The distance between the grid center and the fixed end is 10 mm, while the distance between the grid center and the point at which the load is applied is 300 mm. The conduit is oriented so that the sensor is parallel to the ground. The resistance is measured by a multimeter controlled by a custom VI and the deformation of the conduit surface including the sensor is measured by a digital image correlation (DIC) system, Aramis Adjustable produced by GOM (Braunschweig, Germany). The DIC system is one of the most currently used standard tools in the field of experimental solid mechanics [44] and consists of two high-resolution cameras, two LED lightings fixed on a stable support and a powerful software for the image processing. The illuminated surface is spray painted for the deposition of an appropriate speckle pattern (black speckles over a light surface). The deformation of the recorded images reflects the mechanical deformation of the conduit surface. Considering the image where here no external loads are applied at the conduit end as a reference image, for each image the software calculates the displacement of each subset of black dots with respect to the reference image and therefore the surface strain can be easily obtained. The system was calibrated according to the field of view of the system. The DIC system sends the trigger signal to the multimeter at each image acquisition and the multimeter takes a fixed number of samples. The sample rate of the multimeter is 2.5 Hz. The image acquisition is controlled by the operator according to the type of test. The two systems are controlled by dedicated software executed on a notebook which collects and stores the data measurements. 

Three different bending histories were applied on different conduits equipped with the printed strain gauge. The first test allowed us to obtain the calibration curve by applying different weights and the measurements are taken in steady-state conditions. In the second test, the application of different weights was repeated five times and, in the final test, the resistance signal was recorded for 120 seconds after applying the load.

#### 3.3.1. Strain Test on Bare Sensors

**Figure 9:**
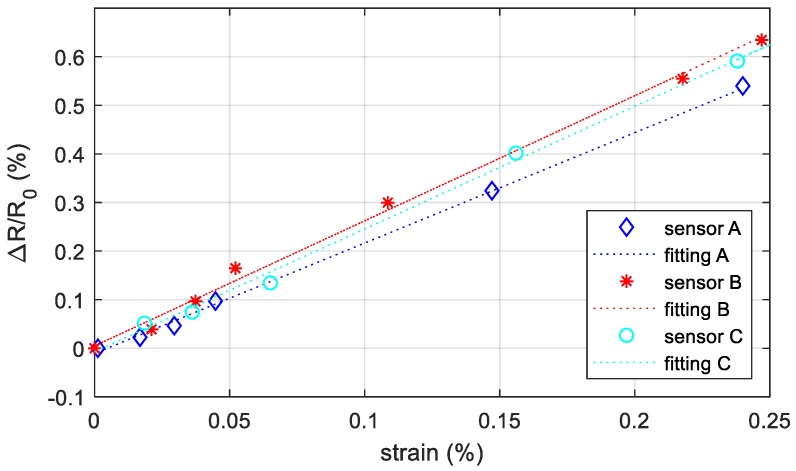
Resistance change with respect to the starting resistance (no external loads) versus the measured strain in the area including the sensor for three conduits equipped with own strain gauge. The points are the measurements data, while the dotted line is the best-fit line using the LLS method.

We analyzed three different sensors printed on three PVC conduits with the same characteristics by applying five weights at the free end. We limited the strain level to 0.25% to stay inside the linear elastic region of the PVC and to prevent plastic deformations. The elaborated results are shown in Figure 9. *R*_0_ represents the resistance of the sensor just before applying the weight and *R* is the resistance after applying the load and waiting a stable signal. In the plot each point is the ratio between the *R-R*_0_ *(ΔR)* and *R*_0_ with respect to the strain measured by the DIC system. The relationship between the resistance ratio and the strain could be considered linear and the gauge factor of sensor A, B, and C is 2.27, 2.57, and 2.53, respectively, and it corresponds to the slope of the best-fit line using the LLS method. 

**Figure 10:**
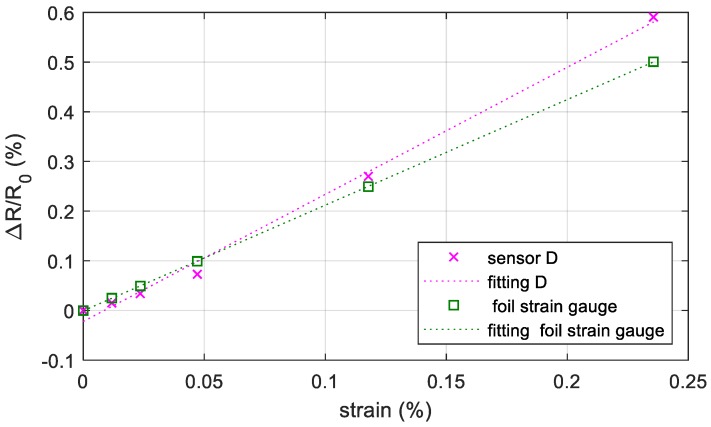
Resistance change with respect to the starting resistance (no external loads) versus the measured strain in the area including the sensor for the printed sensor and for the commercial sensor. The points are the measurements data while the dotted line is the best-fit line using the LLS method.

In order to compare the behavior of the commercial sensor and the printed sensor in the same conditions, a commercial foil strain gauge (commercialized by RS PRO and 2 mm long) [45] was mounted next to the printed strain gauge following the manufacturer’s recommendations. This common and well-known problem did not occur by printing the sensors directly on the surface as proposed. The results are shown in Figure 10; the gauge factor of the printed sensor and of the commercial sensor is 2.55 and 2.12, respectively. 

**Figure 11:**
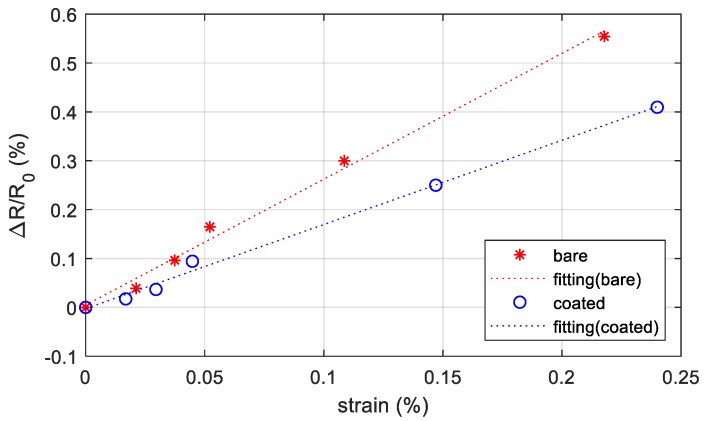
Resistance change with respect to the starting resistance (no external loads) versus the measured strain in the area including the sensor without protection (bare) and with protective layer (coated). The points are the measurements data, while the dotted line is the best-fit line using the least square method.

A protective layer over the printed conductive pattern is often suggested to preserve the sensor and to reduce the influence of external factors (temperature, humidity, etc.) on the sensor response. We covered the printed sensor with SG250 and we applied the same bending history. The results are shown in Figure 11.

The coated layer reduces the sensor response applying the same level of deformation, and this agrees with the literature [46,47]. In this case, the gauge factor of the coated sensor decreases (1.72), but the linearity of the response is preserved.

The results suggest that the fabrication process of the strain gauge ensures good repeatability; indeed, the maximum resistance deviation from the average resistance (71 Ω) calculated on 20 samples was 7 Ω. Considering the relationship between ΔR/R_0_ and the strain, the maximum deviation of the output signal value from the reference straight line is 0.025% and the response can be considered linear as for the commercial foil strain sensor, also when the sensor is encapsulated. 

#### 3.3.2. Cyclic Loading-unloading Test on Printed Strain Gauge Sensors

**Figure 12:**
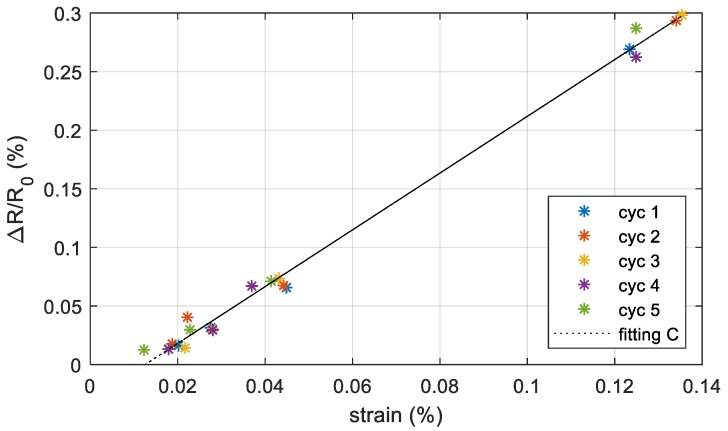
Resistance change with respect to the starting resistance (no external loads) versus the measured strain in the area including the sensor under cyclic loading. The points are the measurements data for each cycle (loading-unloading) while the dashed line is the best-fit line using the least square method.

We tested the sensors by applying cyclically incremental levels of stress in order to define the dynamic behavior of the sensors. The test consists of applying and removing the same load five times. We selected four levels of loads and we waited ten seconds after applying or removing the load. The results obtained on the sensor C (Figure 9) are shown in Figure 12. In these conditions, the gauge factor is 2.42. We obtained a lower gauge factor with respect to the strain test since in the cyclic test the measurements are taken only after 10 seconds from the application of the load, while in the strain test the waiting time was higher than 120 seconds. As explained in Section 3.3.3, the resistance increases slightly over time even under constant stress. The maximum deviation of the output signal value from the reference straight line is 0.017%.

#### 3.3.3. Resistance Response over Time

**Figure 13:**
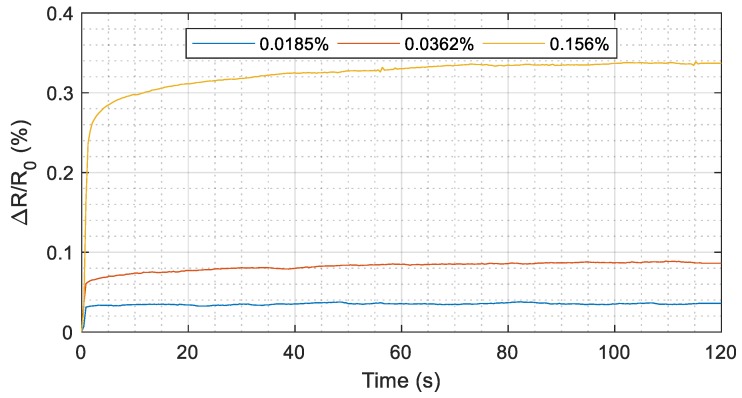
Resistance change with respect to the starting resistance (no external loads) over time by applying different stress levels. The corresponding strain measured at 120th second is reported in the legend for the three cases.

Finally, we tested the resistance response over time, focusing on the resistance change under constant stress. We applied three different values of load and we measured the resistance and the strain for 120 seconds by using the experimental setup described in Section 3.3. The percentage change in resistance (Δ*R*/*R*_0_) over time is shown in Figure 13 for each value of load. The final strain measured by the optical system in the three cases is reported in the legend. The percentage change in resistance (Δ*R*/*R*_0_) reaches the 90% of its final value after 1.2 s for a final strain of 0.0185 % (blue line) and after 12 s in the other two cases. ΔR/R_0_ settles to within 2% of the final value after 70 seconds for strains of 0.036% and 0.0156%. This electrical behavior is partially explained by the mechanical properties of the substrate. In every applied stress, rigid PVC conduits show little creep during time; for example, in the case of the maximum applied stress (yellow line) the strain measured by the optical system at 1, 60, and 120 seconds is 0.120%, 0.145%, and 0.156 %, respectively. The resulting ratio between the sensor response (Δ*R*/*R*_0_) and the measured strain is 2.04, 2.22, 2.16 at 1, 60, and 120 seconds, respectively; these results confirm that the sensor output is able to follow the change in strain. Other mechanical and electrical phenomena on the printed layer affect the change of Δ*R*/*R*_0_ under constant stress and could be detected through an ongoing investigation.

## 4. Conclusions

In this work, we propose a new manufacturing process for printing silver-based strain sensors directly on 3D surfaces. We adopted APJ for selectively depositing a conductive ink on 3D plastic surfaces and photonic sintering for drying and curing the printed layers. To prove the feasibility of the method, we designed a strain gauge with conventional geometry on a plastic conduit; we deposited two layers of silver ink on a PVC conduit and we defined the best setting for the photonic sintering machine. We also evaluated conductivity, maximum thickness, and width of the deposited silver lines that are respectively 40.0 μΩ∙cm, 12.87 μm, and 118 μm. We also evaluated the TCR (0.150 %/°C) for compensating the thermal effect on resistance values. Finally, we manufactured a strain gauge directly on a PVC conduit and we evaluated the gauge factor with respect to a commercial foil strain gauge. The two gauge factors are comparable. In order to protect the printed layer, we also deposited a rubber silicon, which reduced the gauge factor of 30%. The results confirmed that the combination of AJP and photonic sintering is a valid method to produce strain sensors on non-conventional surfaces (non-planar and made of temperature-sensitive materials) and pave the way to fabricate, customize, and integrate sensors and electronics on 3D and temperature-sensitive surfaces using photonic sintering and aerosol jet printing, a versatile technique regarding the variety of functional inks and substrates, the resolution of the printed geometry and the print size.

## References

[1] Wu W. (2017). Inorganic nanomaterials for printed electronics: A review. Nanoscale.

[2] Cruz S.M.F., Rocha L.A., Viana J.C., Rackauskas S. (2018). Printing Technologies on Flexible Substrates for Printed Electronics. Flexible Electronics.

[3] Salaoru I., Maswoud S., Paul S. (2019). Inkjet Printing of Functional Electronic Memory Cells: A Step Forward to Green Electronics. Micromachines.

[4] Cano-Raya C., Denchev Z.Z., Cruz S.F., Viana J.C. (2019). Chemistry of solid metal-based inks and pastes for printed electronics—A review. Appl. Mater. Today.

[5] Khan S., Lorenzelli L., Dahiya R.S. (2015). Technologies for Printing Sensors and Electronics over Large Flexible Substrates: A Review. IEEE Sens. J..

[6] Zhang F., Tuck C., Hague R., He Y., Saleh E., Li Y., Sturgess C., Wildman R. (2016). Inkjet printing of polyimide insulators for the 3D printing of dielectric materials for microelectronic applications. J. Appl. Polym. Sci..

[7] Irimia-Vladu M., Głowacki E.D., Voss G., Bauer S., Sariciftci N.S. (2012). Green and biodegradable electronics. Mater. Today.

[8] Choudhary R.B., Kandulna R., Majumder M., Mandal G. (2018). Electronics with Plastics, Foils and Fabrics: The Ensuing Flexible and Hybrid Electronics. COJ Electron. Commun..

[9] Yang K., Meadmore K., Freeman C., Grabham N., Hughes A.-M., Wei Y., Torah R., Glanc-Gostkiewicz M., Beeby S., Tudor J. (2018). Development of User-Friendly Wearable Electronic Textiles for Healthcare Applications. Sensors.

[10] Al-Shamery K., Raut N.C. (2018). Inkjet printing metals on flexible materials for plastic and paper electronics. J. Mater. Chem. C.

[11] Rajan K., Garofalo E., Chiolerio A. (2018). Wearable Intrinsically Soft, Stretchable, Flexible Devices for Memories and Computing. Sensors.

[12] Khan S., Ali S., Bermak A. (2019). Recent Developments in Printing Flexible and Wearable Sensing Electronics for Healthcare Applications. Sensors.

[13] Feron K., Lim R., Sherwood C., Keynes A., Brichta A., Dastoor P.C. (2018). Organic Bioelectronics: Materials and Biocompatibility. Int. J. Mol. Sci..

[14] Castro H., Correia V., Pereira N., Costab P., Oliveiraa J., Lanceros-Méndez S. (2018). Printed Wheatstone bridge with embedded polymer based piezoresistive sensors for strain sensing applications. Addit. Manuf..

[15] Saengchairat N., Tran T., Chua C.-K. (2017). A Review: Additive Manufacturing for Active Electronic Components. Virtual Phys. Prototyp..

[16] Wang X., Guo W., Zhu Y., Liang X., Wang F., Peng P. (2018). Electrical and Mechanical Properties of Ink Printed Composite Electrodes on Plastic Substrates. Appl. Sci..

[17] Capponi A., Sorger U., Kliazovich D., Fiandrino C., Franck C., Bouvry P. (2017). Assessing Performance of Internet of Things-Based Mobile Crowdsensing Systems for Sensing as a Service Applications in Smart Cities. Proceedings of the 2016 IEEE International Conference on Cloud Computing Technology and Science (CloudCom).

[18] Falco A., Salmerón J.F., Loghin F.C., Lugli P., Rivadeneyra A. (2017). Fully Printed Flexible Single-Chip RFID Tag with Light Detection Capabilities. Sensors.

[19] Shahrubudin N., Lee T., Ramlan R. (2019). An Overview on 3D Printing Technology: Technological, Materials, and Applications. Procedia Manuf..

[20] Le Borgne B., Jacques E., Harnois M. (2018). The Use of a Water Soluble Flexible Substrate to Embed Electronics in Additively Manufactured Objects: From Tattoo to Water Transfer Printed Electronics. Micromachines.

[21] Maurizi M., Slavič J., Cianetti F., Jerman M., Valentinčič J., Lebar A., Boltežar M. (2019). Dynamic Measurements Using FDM 3D-Printed Embedded Strain Sensors. Sensors.

[22] Enser H., Kulha P., Sell J.K., Jakoby B., Hilber W., Strauß B., Schatzl-Linder M. (2016). Printed Strain Gauges Embedded in Organic Coatings. Procedia Eng..

[23] Komurlu E., Cihangir F., Kesimal A., Demir S. (2016). Effect of Adhesive Type on the Measurement of Modulus of Elasticity Using Electrical Resistance Strain Gauges. Arab. J. Sci. Eng..

[24] Wang Y., Qiu Y., Ameri S.K., Jang H., Dai Z., Huang Y., Lu N. (2018). Low-Cost, Μm-Thick, Tape-Free Electronic Tattoo Sensors with Minimized Motion and Sweat Artifacts. npj Flex. Electron..

[25] Saada G., Layani M., Chernevousky A., Magdassi S. (2017). Hydroprinting Conductive Patterns onto 3D Structures. Adv. Mater. Technol..

[26] Borgne B., Liu S., Morvan X., Crand S., Sporea R.A., Lu N., Harnois M. (2019). Water Transfer Printing Enhanced by Water-Induced Pattern Expansion: Toward Large-Area 3D Electronics. Adv. Mater. Technol..

[27] Secor E.B. (2018). Principles of aerosol jet printing. Flex. Print. Electron..

[28] Wilkinson N.J., Smith M.A.A., Kay R.W., Harris R.A. (2019). A review of aerosol jet printing—A non-traditional hybrid process for micro-manufacturing. Int. J. Adv. Manuf. Technol..

[29] Grunwald I., Groth E., Wirth I., Schumacher J., Maiwald M., Zoellmer V., Busse M. (2010). Surface biofunctionalization and production of miniaturized sensor structures using aerosol printing technologies. Biofabrication.

[30] Binder S., Glatthaar M., Rädlein E. (2014). Analytical Investigation of Aerosol Jet Printing. Aerosol Sci. Technol..

[31] Efimov A.A., Arsenov P.V., Minkov K.N., Ivanov V.V. (2018). Fabrication of Metallic Lines by Aerosol Jet Printing: Study of the Effect of Substrate Temperature on the Aspect Ratio. Orient. J. Chem..

[32] Clifford B., Beynon D., Phillips C., Deganello D. (2018). Printed-Sensor-on-Chip devices—Aerosol jet deposition of thin film relative humidity sensors onto packaged integrated circuits. Sensors Actuators B Chem..

[33] Di Novo N.G., Cantù E., Tonello S., Sardini E., Serpelloni M. (2019). Support-Material-Free Microfluidics on an Electrochemical Sensors Platform by Aerosol Jet Printing. Sensors.

[34] Maiwald M., Werner C., Zoellmer V., Busse M. (2010). INKtelligent printed strain gauges. Sensors Actuators A Phys..

[35] Niittynen J., Abbel R., Mäntysalo M., Perelaer J., Schubert U.S., Lupo D. (2014). Alternative sintering methods compared to conventional thermal sintering for inkjet printed silver nanoparticle ink. Thin Solid Films.

[36] Saeidi-Javash M., Kuang W., Dun C., Zhang Y. (2019). 3D Conformal Printing and Photonic Sintering of High-Performance Flexible Thermoelectric Films Using 2D Nanoplates. Adv. Funct. Mater..

[37] Norita S., Kumaki D., Kobayashi Y., Sato T., Fukuda K., Tokito S. (2015). Inkjet-printed copper electrodes using photonic sintering and their application to organic thin-film transistors. Org. Electron..

[38] Borghetti M., Cantù E. Preliminary Study on a Strain Sensor Printed on 3D-plastic Surfaces for Smart Devices. Proceedings of the 2019 II Workshop on Metrology for Industry 4.0 and IoT (MetroInd4.0&IoT).

[39] Lamonaca F., Sciammarella P.F., Scuro C., Carni D.L., Olivito R.S. Internet of Things for Structural Health Monitoring. Proceedings of the 2018 Workshop on Metrology for Industry 4.0 and IoT, MetroInd 4.0 and IoT 2018.

[40] Lee G.-Y., Kim M.-S., Yoon H.-S., Yang J., Ihn J.-B., Ahn S.-H. (2017). Direct Printing of Strain Sensors via Nanoparticle Printer for the Applications to Composite Structural Health Monitoring. Procedia CIRP.

[41] Murillo A.M., Tutikian B.F., Ortolan V., Oliveira M.L.S., Sampaio C.H., Gómez P.L., Silva O.L.F. (2019). Fire Resistance Performance of Concrete-PVC Panels with Polyvinyl Chloride (PVC) Stay in Place (SIP) Formwork. J. Mater. Res. Technol..

[42] Liu Z., Lei Q., Xing S. (2019). Mechanical characteristics of wood, ceramic, metal and carbon fiber-based PLA composites fabricated by FDM. J. Mater. Res. Technol..

[43] EN ISO 4624 International Organization for Standardization (ISO) (2016). Paints and Varnishes—Pull-off Test for Adhesion.

[44] Amiot F., Bornert M., Doumalin P., Dupré J.-C., Fazzini M., Orteu J.-J., Poilâne C., Robert L., Rotinat R., Toussaint E. (2013). Assessment of Digital Image Correlation Measurement Accuracy in the Ultimate Error Regime: Main Results of a Collaborative Benchmark. Strain.

[45] RS PRO (1996). Strain Gauges and Load Cells.

[46] Borghetti M., Sardini E., Serpelloni M. Preliminary study of resistive sensors in inkjet technology for force measurements in biomedical applications. Proceedings of the 2014 IEEE 11th International Multi-Conference on Systems, Signals & Devices (SSD14).

[47] Enser H., Sell J.K., Schatzl-Linder M., Strauß B., Hilber W., Jakoby B. (2017). Hysteresis and Material Effects of Printed Strain Gauges Embedded in Organic Coatings. Multidiscip. Digit. Publ. Inst. Proc..

